# A comparison of regional anesthesia techniques for pain management in patients undergoing liver surgery: a network meta-analysis

**DOI:** 10.3389/fmed.2025.1691322

**Published:** 2025-11-28

**Authors:** Ke Sun, Tao He, Huabo Zhou, Haiyu Song, Cheng Zhang, Qichen Cai

**Affiliations:** Department of Hepatobiliary Surgery, Chengdu Second People’s Hospital, Chengdu, Sichuan, China

**Keywords:** liver surgery, regional anesthesia, epidural analgesia, network meta-analysis, resting pain scores, morphine equivalent

## Abstract

**Objective:**

This research aimed to evaluate the pain-relieving effectiveness and practicality of various regional anesthesia approaches in individuals undergoing hepatic procedures.

**Method:**

A total of 10 randomized controlled trials involving 710 patients were included. We considered any article comprising head-to-head evaluations of at least two of the seven focal regional modalities: continuous subcutaneous/local anesthetic wound infusion (CLAI), continuous erector spinae plane block (ESPB), thoracic epidural analgesia (EA), single-shot erector spinae plane block (ESPB), intrathecal morphine (ITM), quadratus lumborum block (QLB), or continuous thoracic paravertebral block (CTPVB). The primary outcome was postoperative pain scores at rest and movement. Secondary outcomes included morphine consumption at 24, 48, and 72 h; duration of inpatient stay; incidence of postoperative nausea/vomiting (PONV); and any adverse events.

**Results:**

For resting pain scores, pairwise meta-analysis of EA compared to CLAI demonstrated no significant difference at 24 h (SMD = −0.71; 95% CI −2.09, −0.67) and at 48 h postoperatively (SMD = −0.13, 95% CI −0.74, −0.48). However, during movement, EA compared to CLAI demonstrated a significant difference at 24 h (SMD = −1.71, 95% CI −2.26, −1.17) and 48 h (SMD = −0.99, 95% CI −1.46, −0.53) postoperatively. This study does not provide conclusions regarding morphine equivalents at 72 h, the incidence of PONV, or the duration of hospital stay due to the absence of pairwise meta-analysis among the included regional anesthesia techniques.

**Conclusion:**

Among the seven regional anesthesia techniques for liver surgery, EA demonstrates strong analgesic efficacy but requires assessment of coagulation risk, while CESPB and QLB showcase a good safety profile but lack sufficient long-term evidence. This study offers comparative insights to guide clinical decisions rather than definite evidence of overall reliability or low risk associated with regional anesthesia.

## Highlights

- Regional anesthesia is effective postoperative analgesia method for liver surgery.
- Epidural anesthesia is effective in reducing pain scores in movement.
- Epidural anesthesia provides the best pain management after liver surgery.
- Epidural anesthesia can be interpreted with caution due to the inherent risks of liver surgery.
- Network meta-analysis can make indirect quantitative comparisons.

## Introduction

Liver surgery is inherently associated with significant trauma and severe pain (1). Despite advancements in minimally invasive techniques, which involve smaller skin incisions and reduced tissue trauma, managing postoperative pain remains a formidable challenge. Poorly managed pain impedes healing and impacts both daily comfort and psychological health (1). Moreover, it significantly increases the risk of developing chronic postsurgical pain (CPSP) (2, 3). The incidence of CPSP can vary widely, ranging from 5 to 50%, thus posing a significant barrier to rapid recovery (4). Therefore, optimizing analgesic methods is essential to facilitate early recovery and rehabilitation in patients undergoing liver surgery.

Regional anesthesia methods have gained significant preference among clinicians and include a variety of techniques such as epidural analgesia (EA), intrathecal morphine (ITM), continuous erector spine plane block (ESPB), quadratus lumborum block (QLB), local anesthetic infiltration (LAI), continuous thoracic paravertebral block (TPVB), and transverse abdominal plane block (TAP) (5–14). Among these, EA is widely recognized as a standard regional analgesia technique for many major abdominal surgeries (13). However, for major hepatectomy, the trade-offs associated with thoracic epidurals warrant scrutiny: resection-triggered coagulopathy can provoke spinal bleeding, and leaving the catheter *in situ* prolongs the period of hematoma risk. Catastrophic neurological events are uncommon, and no organ-specific guidelines exist; therefore, protection depends on adherence to antithrombotic consensus guidelines and screening for postresection clotting derangements (15). While EA can effectively reduce the need for systemic opioid administration and enhance gastrointestinal and immune functions, existing evidence indicates that it is technically challenging to perform and is associated with a range of complications. These complications can vary from pneumothorax to spinal cord injury (1, 13). Additionally, the coagulopathy often seen in liver surgery patients may further restrict the use of EA (1).

The thoracic paravertebral block (TPVB) has emerged as an alternative regional technique in clinical practice. It offers a comparable analgesic profile to EA but with fewer complications, including nausea or pruritus (13). The dispersion of local anesthetics in the paravertebral space allows TPVB to provide relatively extensive analgesic coverage (13). Similarly, intrathecal morphine (ITM) can provide effective postoperative analgesia, comparable to EA. However, its use is limited by various complications: frequent postoperative nausea and vomiting (PONV), pruritus, and, importantly, the potential for delayed respiratory depression (7). Recent studies have demonstrated that the erector spinae plane block (ESPB) can offer promising analgesia for liver procedures, emerging as a safe alternative to the quadratus lumborum block (QLB) (11). However, some studies suggest that ESPB may not significantly reduce opioid demand or pain scores (16). Additionally, the use of transverse abdominal plane block (TAP) and local anesthetic infiltration (LAI) often fails to adequately relieve the visceral pain associated with liver surgery (17).

To address these complexities, we conducted a network meta-analysis (NMA) to determine which regional anesthesia technique offers superior analgesic efficacy and safety to patients undergoing liver surgery. Conventional meta-analysis, when evaluating three or more treatments, is limited to pairwise comparisons of benefit and harm, assessing only one pair at a time. Additionally, the limited stock of direct, head-to-head randomized studies for numerous therapies creates a major hurdle for the standard two-treatment synthesis model. In contrast, NMA enables indirect statistical comparisons even when head-to-head trials are absent. The analysis yields actionable insights into relative treatment merit and highlights the most effective and least hazardous options. This study sought to rank pain-control strategies for hepatic resections and identify a best-practice regimen to guide clinicians.

## Methods

This systematic review and meta-analysis were planned, executed, and reported in full accordance with the Preferred Reporting Items for Systematic Reviews and Meta-Analyses (PRISMA) statement and the Assessing the Methodological Quality of Systematic Reviews (AMSTAR) checklist (18). To enhance transparency, the review protocol was submitted to and accepted by the International Platform of Registered Systematic Review and Meta-analysis Protocols (INPLASY) ahead of any data extraction, receiving the unique registration identifier INPLASY202250090.

### Search strategy

A comprehensive search of PubMed, Embase, the Cochrane Library, and Web of Science was performed from database inception to February 2022 to identify eligible randomized controlled trials. The structured query, detailed in Supplementary Table S1, paired free-text and Medical Subject Headings (MeSH) terms. Because the ESPB is an emerging technique first described in 2016, we extended the hunt to conference abstracts, theses, and trial registries through targeted grey-literature hand searching.

### Study selection criteria

Two reviewers worked separately to run the search and screen titles, abstracts, and full texts; conflicts were settled by consensus or, when needed, by a third adjudicator. We admitted every published, peer-reviewed randomised controlled trial (RCT) that gauged pain control provided by any form of regional anesthesia in adults undergoing hepatic resection, transplantation, or other open or laparoscopic liver procedures. The PICOS criteria were as follows: Patients (P): adult patients scheduled for elective liver surgery; Intervention (I): EA, CLAI, ITM, ESPB, CESPB, QLB, or CTPVB; Comparison (C): other analgesic methods; Outcome measures (O): pain intensity at rest and during movement 24 and 48 h postoperatively served as the primary endpoint. Secondary variables captured total opioid use, incidence of postoperative nausea and vomiting, any adverse events, duration of hospital stay, and patient-reported satisfaction scores. The study design (S) followed was RCT. No language restrictions were applied; however, case reports, truncated trials, and conference abstracts lacking adequate design or outcome details were discarded. Moreover, the included regional anesthesia techniques were classified based on two core principles: the clinical relevance to liver surgery and methodological uniqueness of analgesic delivery. This classification was intentionally retained (rather than merging similar-sounding techniques) to ensure alignment with the study design. After the initial electronic search, two reviewers independently screened titles and abstracts to identify potentially eligible studies, resolving any disagreements through discussion until consensus was reached.

### Data extraction and outcome measures

#### Data extraction

Two reviewers independently drew participant baseline data, including age, sex, comorbidities, and anesthetic interventions, from all relevant text, tables, and figures. After cross-checking the parallel extractions, methodological rigor was assessed using the Cochrane risk-of-bias instrument (19). The following domains were evaluated: random sequence generation, allocation concealment, masking of patients and assessors, follow-up completeness, and selective reporting. Each domain was scored as “high” or “low” risk of bias.

#### Outcome measures

Primary endpoints were pain scores at rest and during movement at 24, 48, and 72 h postoperatively. Secondary endpoints included cumulative morphine use at 24, 48, and 72 h; duration of inpatient stay; postoperative complications (nausea/vomiting, urinary retention, and pruritus); and patient-reported satisfaction. Hypotension, dizziness, and other infrequent events were excluded, as few trials provided extractable data.

### Statistical analysis

All quantitative analyses for this network meta-analysis were performed using STATA 17.0 (Stata Corp, College Station, TX) and RevMan 5.4 (Copenhagen: Nordic Cochrane Centre). For dichotomous endpoints, we summarized the contrast as an odds ratio, while for continuous endpoints, we used the standardized mean difference. The estimate of each effect was accompanied by its 95% confidence interval (20). If different data records were used, the formula described by Hozo et al. (21) was employed. Whenever outcomes were available only in graphical form, data were extracted using WebPlotDigitizer (22). All perioperative opioid doses were converted to intravenous morphine equivalents with the previously adopted standard calculator (23). Methodological quality was assessed using RevMan 5.4 and summarized in a bias-risk diagram. Network geometry, forest, rank-probability, and funnel plots, along with their inferential tests, were analyzed in STATA 17.0 (24). Between-study inconsistency was quantified using I^2^ statistic; values above 50% prompted a random-effects synthesis, whereas values of 50% or below justified a fixed-effects model (25). When notable heterogeneity emerged, analysis relied on a random-effects framework, and in its absence, a fixed-effects specification was adopted. Consistency across direct and indirect evidence was evaluated using node-splitting; a *p*-value ≥ 0.05 was statistically significant between the two sources (26). A random-effects pairwise meta-analysis pooled trials that reported identical endpoints and treatments. In STATA 17, if the plot quantified the 95% CI and inconsistency factor for every closed loop in the network, when the lower bound approached or crossed zero, direct and indirect estimates were deemed highly coherent. The Surface Under the Cumulative Ranking (SUCRA) curve converts each treatment’s rank into a cumulative probability, where larger values signify better outcomes. Publication bias was evaluated using funnel plots.

## Results

### Study selection and characteristics

**Figure 1:**
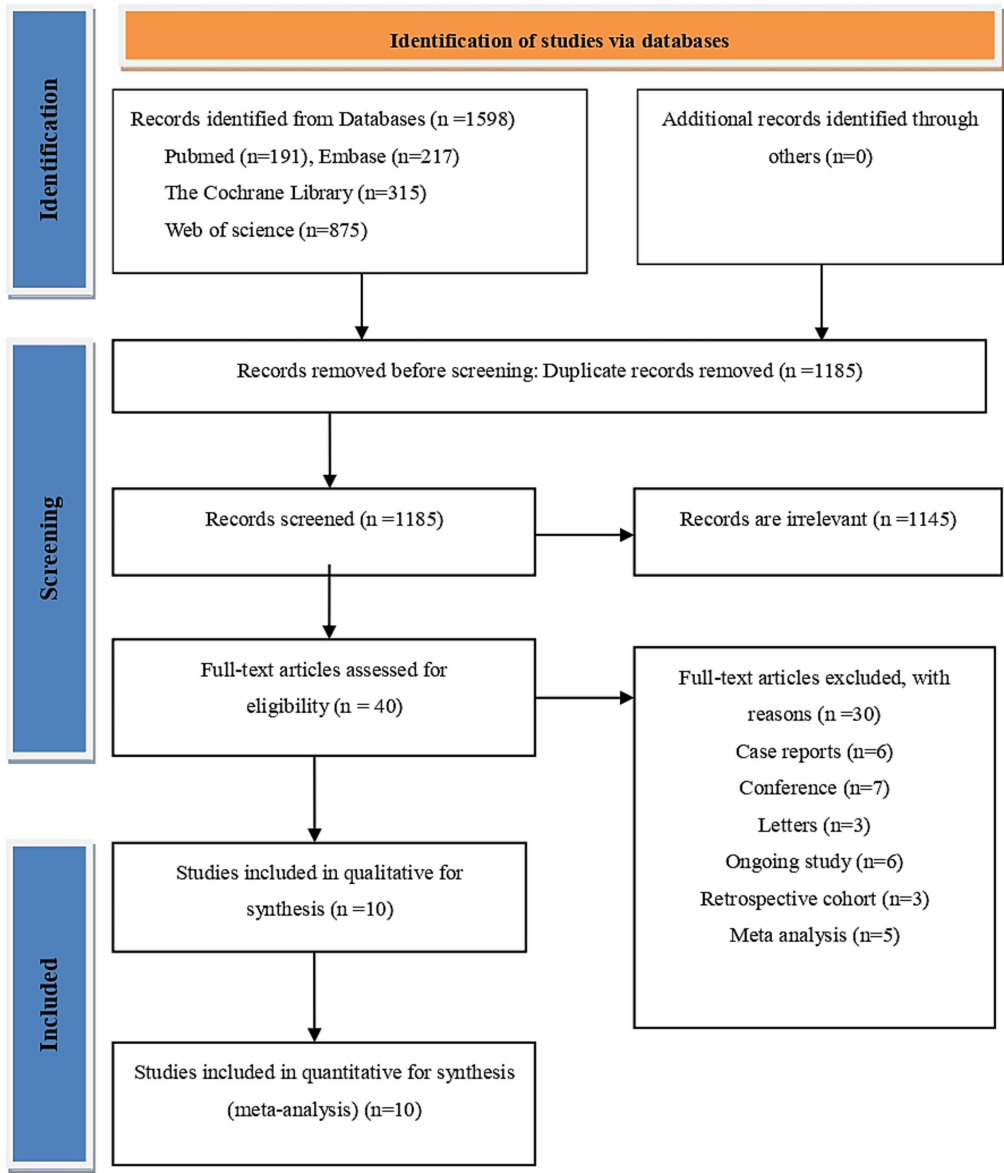
PRISMA flow chart for the study selection process and provides reasons for exclusion for the records screened. PRISMA, preferred reporting items for systematic reviews and meta-analysis.

Four electronic databases were systematically searched, including PubMed (*n* = 191), Embase (*n* = 217), the Cochrane Library (*n* = 315), and Web of Science (*n* = 875). A total of 1,598 references were initially identified through this search strategy. EndNote X9 (Clarivate, London, UK) plus was used for the removal of duplicates, followed by sequential screening of titles, abstracts, and full texts, which resulted in 10 eligible randomized trials comprising 710 participants (Figure 1). These 10 reports are summarized in Table 1.

**Table 1:** Overview and characteristics of included studies’ in patients undergoing patients undergoing liver surgery.

Study	Year	Regime	No	Age	Anesthesia	Intervention	Surgery	Gender (M/F)	Analgesic modality	Pain scale	Rest/ Movement	Outcomes	ASA
Bell et al. (4)	2019	UK	83	67.3 (25–85)vs. 65 (24–79)	GA	(1)(2)	Open liver resection	29/12vs27/12	PCA	VAS	NA	[1][2][4][5][6]	I - II
De Pietri et al.(6)	2006	Italy	50	NA	GA	(1)(3)	Open liver resection	NA	PCA	VAS	Rest	[1][2][3][4][5]	I - II
Hughes et al. (5)	2015	UK	93	62.6 ± 11.1vs62.8 ± 12.1	GA	(1)(2)	Open liver resection	29 /15vs28/21	PCA	VAS	Both	[1][2][3][5]	I - II
Kang et al. (7)	2019	South Korea	54	36.5 ± 10.5vs32.9 ± 12.4	GA	(3)(4)	Laparoscopic hepatectomy	20/7vs12/15	PCA	NRS	Rest	[1][2][3][5]	I - II
Kang et al. (9)	2021	South Korea	60	37.4 ± 12.1 vs38.6 ± 13.1	GA	(3)(5)	Laparoscopic hepatectomy	13/16vs18/12	PCA	NRS	Rest	[1][2][3][4][5][6][7]	I - II
Kang et al. (11)	2021	South Korea	85	52.7 ± 11.9vs52.6 ± 9.4	GA	(4)(6)	Laparoscopic Liver Resection	27/15vs30/13	PCA	NRS	Rest	[1][2][3][5][4][5][6][7]	I - III
Koea et al. (8)	2009	New Zealand	100	60(23–79)vs61(28–83)	GA	(1)(3)	Liver Resection	24/26vs27/23	NA	NA	NA	[1][2][5]	NA
Lee et al. (10)	2013	South Korea	40	30.5 ± 8.7 vs35.6 ± 11.0	GA	(2)(3)	Liver Transplantation	12/7vs15/6	PCA	VAS	Rest	[1][2][5][7]	I - II
Revie et al. (12)	2012	UK	65	60 (23–85)vs60 (39–84)	GA	(1)(2)	Liver resection	17/16vs19/12	NA	—	Both	[1][2][3][4][5]	I - III
Schreiber et al. (13)	2016	US	80	61.5 ± 13.7 vs56.2 ± 12.9	GA	(1)(7)	Open liver resection	21/20vs24/15	NA	VRS	Rest	[1][2][3][4][5]	I - II

Intervention: 1. EA, Epidural analgesia; 2. CLAI, Continuous local anesthetic infiltration; 3. ITM, Intrathecal Morphine; 4. ESPB, Erector spinae plane block; 5. CESPB, Continuous ESPB; 6. QLB, Quadratus lumborum block; 7. CTPVB, Continuous thoracic paravertebral block.

Outcomes: 1. Pain scores, 2. Postoperative morphine Equivalent (mg), 3. First time require analgesics, 4. PONV, Postoperative nausea and vomiting; 5. Adverse events, 6. Length of hospital stay, 7. Patients satisfaction. Data are present as mean ± SD or median (IQR).

VAS, Visual analogue pain scores; NRS, Numeric rating scale; VRS, Verbal Rating Scale; GA, General anesthesia; PCA, Patient-controlled analgesia.

### Risk bias evaluation

**Figure 2:**
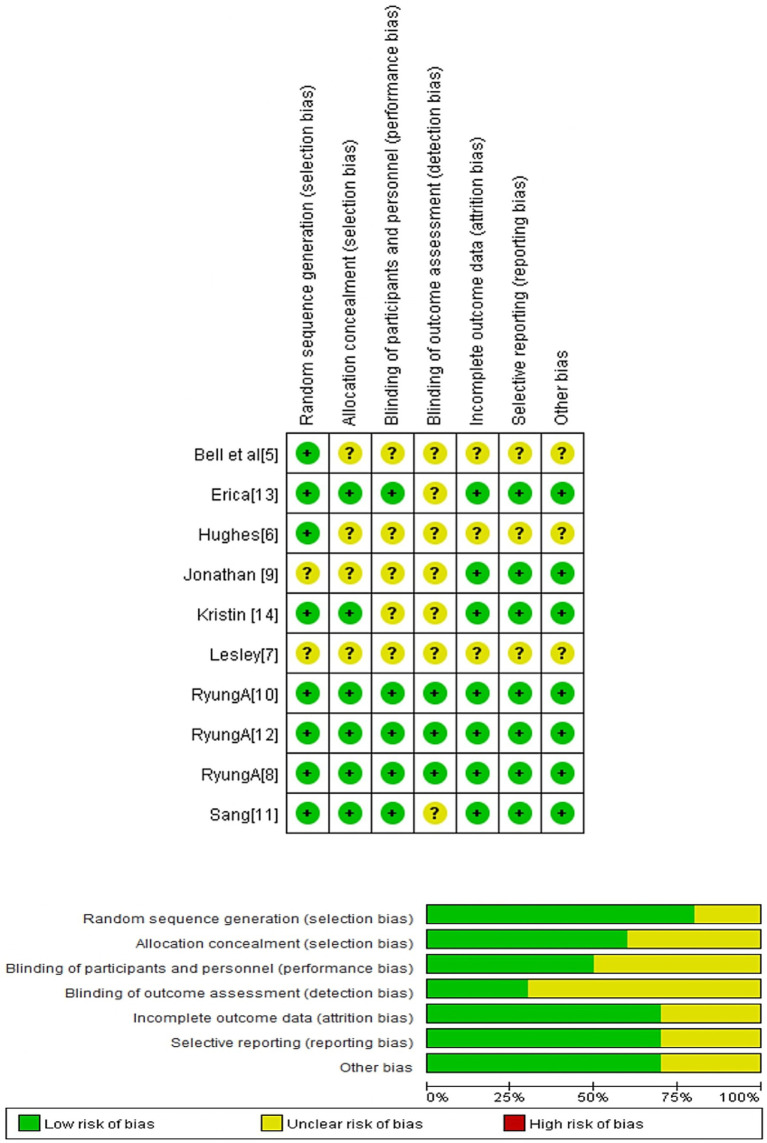
Risk of bias summary: Authors’ judgments about each risk of bias item for the included study.

Figure 2 presents the risk of bias assessment for the 10 included studies. According to the Cochrane risk of bias tool (19), three studies showed a relatively low risk of bias across critical domains (7, 9, 11). In contrast, the remaining 7 studies exhibited a moderate to high risk of bias (4–6, 8, 10, 12, 13).

### Result merging

**Figure 3:**
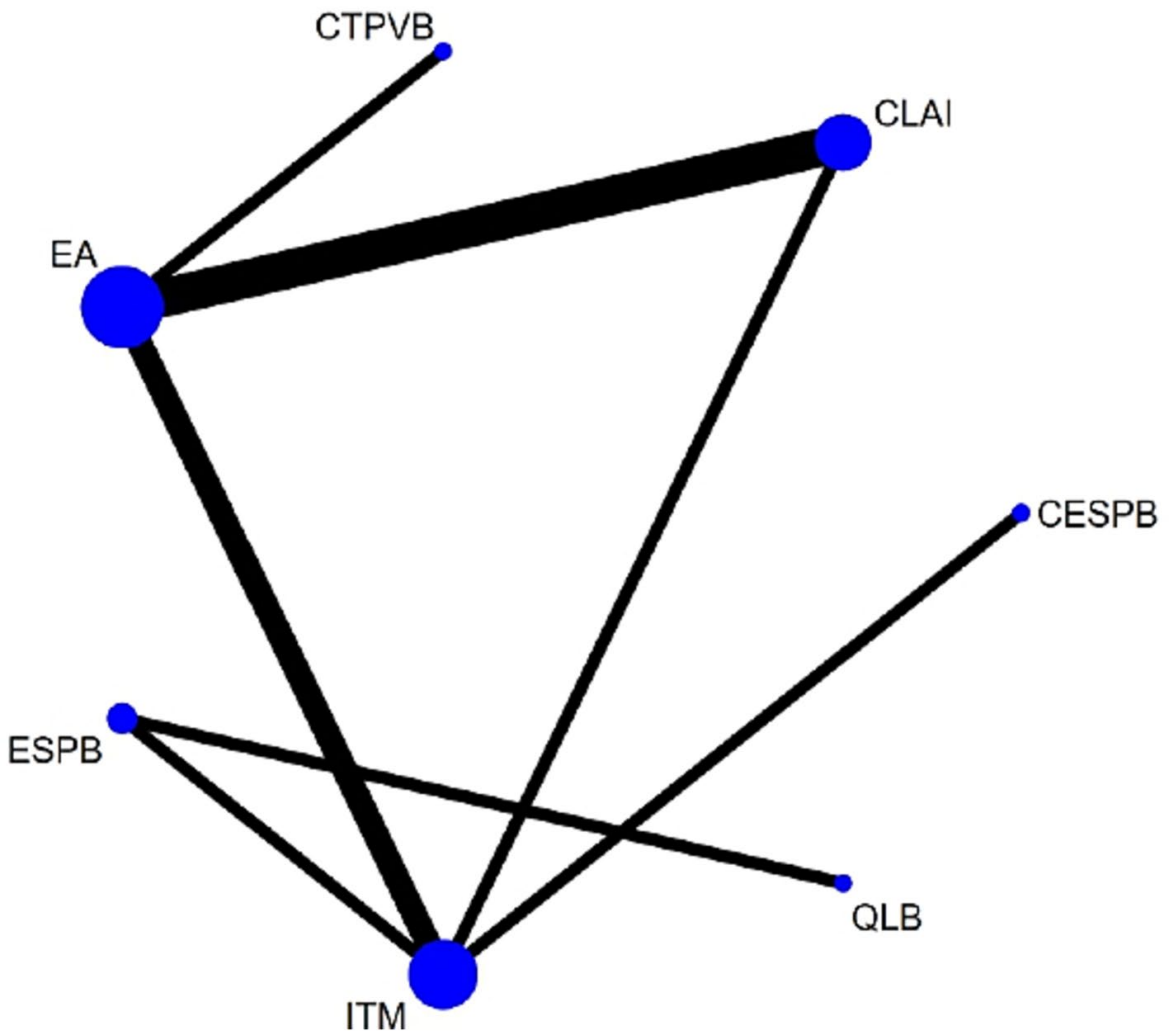
Network plot of available interventions comparisons for response rates. Size of node is proportional to number of patients randomized to reach interventions. Line width is proportional to the number of randomized controlled trials comparing each pair of interventions. EA, Epidural analgesia; CLAI, Continuous local anaesthetic infiltration; ITM, Intrathecal Morphine; ESPB, Erector spinae plane block; CESPB, Continuous ESPB; QLB, Quadratus lumborum block; CTPVB, Continuous thoracic paravertebral block.

Figure 3 illustrates the intervention network formed by the included trials. Seven regional analgesia methods were evaluated in the current network meta-analysis: EA, CLAI, ITM, ESPB, CESPB, QLB, and CTPVB. A total of three studies compared EA with CLAI (4, 5, 12); two studies compared EA with ITM (6, 8); and three studies assessed ITM against CLAI, ESPB, and CESPB, respectively (7, 9, 10). One study compared ESPB with QLB (11), and another study evaluated EA against CTPVB (13).

### Outcome measures

Supplementary Figure S1 presents the complete evidence-network map for each prespecified primary and secondary endpoint, which illustrates how the 10 included RCTs interconnect through shared comparators and outcome definitions. For every outcome measure, homogeneity between direct and indirect treatment effects was confirmed: all network nodes maintained internal consistency, and no statistically significant inconsistency loops were detected (Supplementary Table S2).

**Figure 4:**
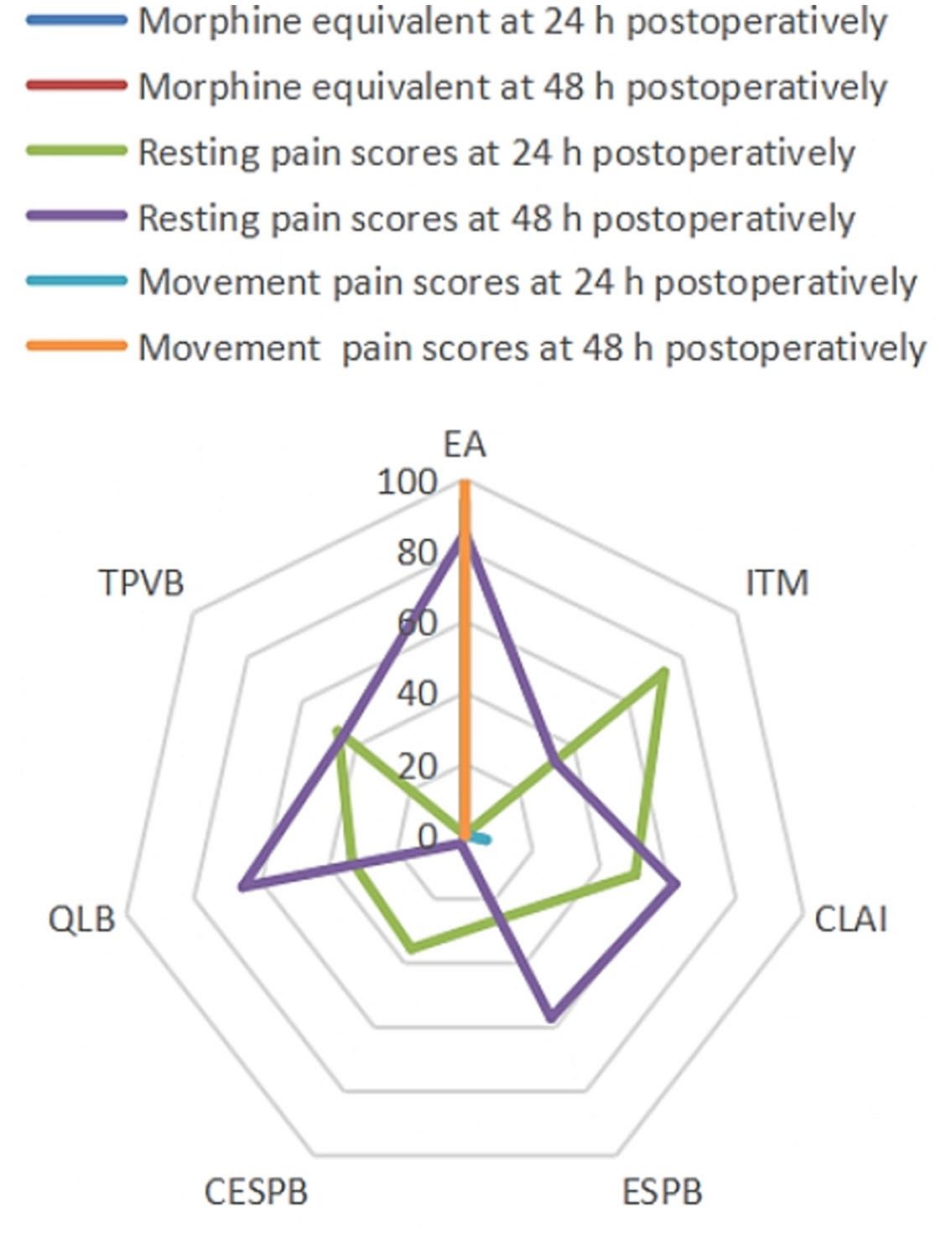
Values of SUCRA for all outcomes. SUCRA, Surface under the cumulative ranking curve; EA, Epidural analgesia; ITM, Intrathecal morphine; CLAI, Continue local anesthetic infiltration; ESPB, Erector spinae plane block; CESPB, Continue erector spinae plane block; QLB, Quadratus lumborum block; CTPVB, Continuous thoracic paravertebral block.

To explore small-study effects, comparison-adjusted funnel plots were constructed (Supplementary Figure S1); visual inspection and Egger’s regression test provided no indication of asymmetry (*p* > 0.10 for each outcome), thereby suggesting the absence of meaningful publication bias. The relative hierarchy of interventions, presented as SUCRA values and mean ranks with 95% confidence intervals, is provided in Supplementary Table S3. Moreover, Figure 4 demonstrates the SUCRA values of the seven regional anesthesia techniques across all prespecified primary and secondary efficacy outcomes, providing a visual summary of their relative effectiveness in postoperative pain control and opioid-sparing following liver surgery. EA maintained top rankings for all pain and opioid-related endpoints, and QLB showed improved efficacy in 48 h resting pain compared to 24 h, suggesting a potential difference in analgesia duration between techniques. Collectively, these SUCRA-based rankings provide a comprehensive, patient-centered reference to guide clinicians in selecting regional anesthesia strategies, optimizing both postoperative pain control and opioid-sparing for individual liver surgery patients. Furthermore, the corresponding forest plot visualizes pooled effect sizes along with their associated prediction intervals for all primary and secondary outcomes (Supplementary Figure S3). To ensure methodological transparency for critical analysis of the study findings and to facilitate accessibility for future updates or secondary analyses, all supplementary materials (Supplementary Tables S1–S3 and Supplementary Figures S1–S3) are provided as separate downloadable files.

### Resting pain scores at 24 and 48 h postoperatively

In total, seven trials captured pain scores at 24 and 48 h postoperatively, covering a set of seven distinct interventions: EA, CLAI, ITM, ESPB, TPVB, CESPB, and QLB. Pairwise meta-analysis comparing EA with CLAI postoperatively revealed no significant difference at 24 (SMD = −0.71, 95% CI −2.09, −0.67) and 48 h (SMD = −0.13, 95% CI −0.74, −0.48).

### Movement pain scores at 24 and 48 h postoperatively

Two studies reporting movement pain scores at 24 and 48 h postoperatively evaluated two interventions: EA and CLAI. EA compared to CLAI demonstrated a significant difference postoperatively at 24 (SMD = −1.71, 95% CI −2.26, −1.17) and 48 h (SMD = −0.99, 95% CI −1.46, − 0.53) (see Table 2).

**Table 2:** Pair-wise meta-analysis comparing intravenous pain scores (visual analog scale scores or numerical rating scale) after 24 and 48 h, and postoperative vomiting and nausea.

Comparison	Included studies	Pair-wise meta-analysis	*p* value	I^2^, %
		SMD (95% CI)		
Resting pain at 24 h and 48 h				
EA vs. CLAI	2	−0.71 [−2.09, 0.67]	0.31	81%
Resting pain at 48 h				
EA vs. CLAI	2	−0.13 [−0.74, 0.48]	0.67	0%
Movement pain at 24 h				
EA vs. CLAI	2	−1.71 [−2.26, −1.17]	0.001	24%
Movement pain at 48 h				
EA vs. CLAI	2	−0.099 [−1.46, −0.53]	0.001	0%

### Morphine equivalent at 24, 48, and 72 h postoperatively

Five studies reported postoperative morphine equivalents at 24 and 48 h, evaluating the following interventions: EA, CLAI, ITM, ESPB, CESPB, and QLB. A total of four trials investigated postoperative morphine equivalent at 72 h, assessing EA, CLAI, ITM, ESPB, and QLB. There is no pairwise meta-analysis of the included regional anesthesia methods.

### The PONV and the length of hospital stay

Postoperative nausea and vomiting (PONV) were documented in three trials that together evaluated four regional modalities—intrathecal morphine (ITM), erector spinae plane block (ESPB), continuous erector spinae plane block (CESPB), and quadratus lumborum block (QLB), while hospital length of stay was reported in a separate set of three trials. Because each outcome was dispersed across non-overlapping comparisons with no common control arm, the resulting evidence network was too sparse to support reliable pairwise or network meta-analysis, and thus, quantitative synthesis for PONV and length of stay could not be performed.

### The details of liver surgery

A total of 10 studies were included in the current review (4–13): six studies investigated open liver resections (4–6, 8, 12, 13); three studies investigated laparoscopic hepatectomy (7, 9, 11); and one study investigated liver transplantation (10), as shown in Table 3. In most studies, right subcostal with midline incision (5–13) was employed, with the incision choice dictated by surgeons only in one study (4). Most studies focused on the postoperative recovery indicators, including pain scores, opioid consumption, quality of recovery, and postoperative complications; only three studies recorded the postoperative coagulation function (5, 8, 13).

**Table 3:** The details of liver resection of included studies in the current review.

Study	Surgery	Incision	Coagulation function	Hepatic resections
Bell et al. (4)	Open liver resection	Decided by surgeons	NA	Major: ≥3, Minor: <3
De Pietri et al. (6)	Open liver resection	Right subcostal incision with mid-line	Postoperative INR and platelet count	Major: ≥3, Minor: unilateral and bilateral segmentectomies
Hughes et al. (5)	Open liver resection	Right subcostal incision with mid-line	NA	Major: ≥3, Minor: <3
Kang et al. (7)	Laparoscopic hepatectomy	Right subcostal incision with mid-line	NA	NA
Kang et al. (9)	Laparoscopic hepatectomy	Right subcostal incision with mid-line	NA	NA
Kang et al. (11)	Laparoscopic Liver Resection	Right subcostal incision with mid-line	NA	NA
Koea et al. (8)	Open liver resection	Right subcostal incision with mid-line	Postoperative continues 5 days INR<1.5	Major: ≥4, Minor: <4
Lee et al. (10)	Liver transplantation	Right subcostal incision with mid-line	NA	NA
Revie et al. (12)	Open liver resection	Right subcostal incision with mid-line	NA	NA
Schreiber et al. (13)	Open liver resection	Right subcostal incision with mid-line	Postoperative INR and platelet count	NA

INR, International Normalized Ratio; NA, Not applicable.

Furthermore, several studies evaluated hepatic resection and classified it as a major or minor segment resection (5, 6, 8). De Pietri et al. (6), Bell et al. (4), and Hughes et al. (5) classified liver resections as major if they involved more than three segments, and minor if they involved fewer than three segments. Jonathan and colleagues classified resections as major if they included more than four segments, and minor if they involved fewer than four segments (8). Furthermore, De Pietri et al. (6) discovered that patients undergoing major resection had a higher international normalized ratio (INR) than patients undergoing minor resection. However, no significant difference in platelet count was observed between major and minor resections (6).

## Discussion

This NMA encompassed seven interventions for postoperative pain management. The analysis focused on several key aspects: postoperative resting and movement pain scores at 24, 48, and 72 h; postoperative intravenous morphine consumption at 24, 48, and 72 h; postoperative vomiting and nausea; length of hospital stay; and adverse events. The present systematic review and network meta-analysis found that no single intervention was universally superior across all outcomes when managing postoperative pain following liver surgery. Nevertheless, EA emerged as the most effective technique for surgical pain management (6). Network rankings placed EA first for resting VAS at 24 h and 48 h, first for dynamic VAS during coughing or mobilization at the same intervals, and first for cumulative intravenous morphine-sparing at both timepoints. Collectively, these metrics established EA as the most potent of the seven regimens examined for analgesia after hepatic resection. Optimal pain control following liver resection not only blunts postoperative discomfort but also shortens the time to ambulation, lowers the incidence of cardiopulmonary complications, and shortens hospital stay (1). Within the network, we found that epidural anesthesia consistently produced the smallest visual-analogue pain scores at every measured interval and, simultaneously, the smallest cumulative intravenous morphine requirement, indicating that, among the regimens compared, it offers the most complete and opioid-sparing analgesia for patients recovering from hepatic surgery. Favored in hepatic cases, ESPB offers easier execution, a cleaner safety record, and broader ipsilateral thoracic coverage than EA (27). EA, a promising and effective regional technique, is widely used by clinicians, and the study data demonstrated that it provides effective analgesia in liver surgery (1, 6). Conversely, the present NMA revealed that, relative to CLAI, it delivered better results in movement-evoked pain scores (*p* = 0.001). Moreover, the SUCRA values highlighted that EA is superior to CLAI in terms of movement pain scores (7). Bell et al. (4) and Hughes et al. (5) demonstrated that compared with CLAI, EA exhibited no significant differences in PONV, hospital length of stay, or postoperative complications. Additionally, Revie et al. (12) found no significant difference between EA and CLAI in terms of first-mobilization timing or total complication incidence (58.1% vs. 48.5%, *p* = 0.443). However, the pooled evidence comparing EA with CLAI showed both direct and indirect inconsistency, urging guarded inference; this pairwise contrast yielded an inflated effect size, likely reflecting the discrepancy. Furthermore, the results need to be interpreted with caution due to the inherent risk that liver surgery carries and limitations of EA use in the surgery (1). Introduced by Forero et al. in 2016, ESPB is a novel regional block now gaining popularity (28). When the appropriate spinal segment is selected, the technically simple ESPB provides wide dermatomal coverage (T1 to L3) and effectively blocks the trunk segments. Its efficacy has been validated across diverse operative contexts (29–36). This NAM synthesis revealed no trial-level evidence that ESPB outperforms ITM or QLB in terms of 24-h pain scores or postoperative opioid requirements (7, 9, 11). These findings are consistent with those of Kim et al. (16), who reported that ESPB does not reduce postoperative pain scores or morphine consumption in patients undergoing liver surgery. Nevertheless, ESPB reduced the incidence of PONV and pruritus within 24 h compared to ITM (7, 9). Interestingly, a recent study demonstrated that ESPB can offer better pain control than patient-controlled intravenous analgesia (PCIA) and reduce intraoperative and postoperative opioid consumption (32). However, our findings suggest that the optimal concentration and dose of local anesthetics used in ESPB require further investigation (32). Compared to QLB, ESPB demonstrated no significant difference in terms of PONV and other complications (11). Conversely, Fu et al. (37) reported that ESPB provides promising analgesic effects compared to non-block care in these surgeries. Given the current conflicting results regarding ESPB, further studies are warranted.

Coagulopathy significantly increases the risk of spinal canal bleeding or infection, particularly with prolonged catheter placement; in patients with cirrhosis, engorged epidural veins further predispose to needle trauma and bleeding. As catheter removal is associated with approximately 50% of bleeding incidents, this procedure must be performed with utmost caution. Neuraxial block remains a viable and beneficial option for patients undergoing liver resection, particularly when preoperative laboratory evaluations, such as the international normalized ratio (INR), aspartate aminotransferase (AST), and alanine aminotransferase (ALT), are within the normal range. In cases where these parameters are slightly elevated, but the surgical procedure is classified as minor, the use of epidural anesthesia may still be a viable option, provided careful clinical judgment is applied. However, patients with an INR greater than 1.1 and an AST level greater than 50 U/L should be approached with caution, as these values may indicate underlying liver disease (15).

Given the risk of coagulopathy associated with liver surgery, CTPVB has been proposed as an alternative regional analgesic technique. The paravertebral space contains the intercostal nerves, making it an ideal location for blocking nociceptive transmission (38). In the current NMA, one study compared EA with CTPVB and demonstrated that CTPVB could offer comparable analgesia with fewer adverse effects compared to EA. Previous research comparing the analgesic and adverse effect profiles of EA and CTPVB in other types of surgeries demonstrated no significant differences in pain control (39–41). However, further evidence is needed to establish the safety, feasibility, and efficacy of CTPVB in liver surgery.

### Limitations

Several caveats temper these conclusions. First, the evidence is based on only 10 trials, which showed moderate to high heterogeneity, potentially weakening the reliability of the current study’s findings. Second, the liver surgeries included both open incisions and laparoscopic procedures; however, the limited number of trials prevented us from conducting subgroup analyses. Third, the varying concentration of anesthetic drugs and their technical details may have influenced the outcomes. Finally, the limited number of trials and incomplete datasets prevented several planned sensitivity and subgroup analyses. The sample size was inadequate to draw definitive conclusions. Additionally, as ESPB is an emerging technique, it may be subject to publication bias.

## Conclusion

This network meta-analysis provides a comprehensive and comparative evaluation of seven regional anesthesia techniques for postoperative pain management in liver surgery. The findings indicate that epidural analgesia (EA) consistently provides superior analgesic efficacy during the first 24 to 48 h postoperatively, reducing both resting and movement-evoked pain scores, as well as opioid consumption. However, its application must be carefully evaluated for the potential risks associated with coagulopathy, a common concern in liver surgery patients. Emerging techniques, such as continuous erector spinae plane block (CESPB) and quadratus lumborum block (QLB), demonstrate promising safety profiles, particularly in patients where neuraxial techniques may be contraindicated. However, long-term efficacy data remain limited, and additional high-quality randomized trials are required to establish their roles in routine clinical practice. Importantly, no technique was universally superior across all outcomes.

## Data Availability

The original contributions presented in the study are included in the article/Supplementary material, further inquiries can be directed to the corresponding author.
