# Supplementary material for: A comparison of regional anesthesia techniques for pain management in patients undergoing liver surgery: a network meta-analysis
Source: Front Med (Lausanne). 2025 Nov 28;12:1691322. doi: 10.3389/fmed.2025.1691322 (PMC12698550; doi:10.3389/fmed.2025.1691322)
Supplement: Supplementary file 1 [file Table_1.doc]

Supplementary Table 1: Pubmed search terms for searching RCTs

| Set # |  |
| --- | --- |
| 1 | ((((Transversus abdominis plane block[Title/Abstract]) OR (transversus abdominis plane[Title/Abstract])) OR (TAP block[Title/Abstract])) OR (TAP[Title/Abstract])) OR (TAPB[Title/Abstract]) |
| 2 | ****((((Paravertebral block[Title/Abstract]) OR (paravertebral anaesthesia[Title/Abstract])) OR (Paravertebral Blockade[Title/Abstract])) OR (Paravertebral blocks[Title/Abstract])) OR (PVB technique [Title/Abstract])**** |
| 3 | **((((("Nerve Block"[Mesh]) OR (anesthesia conduction[Title/Abstract])) OR (Nerve Blocks[Title/Abstract])) OR (Nerve Blockade[Title/Abstract])) OR (Nerve Blockades[Title/Abstract])) OR (regional anesthesia[Title/Abstract]** |
| 4 | **(((erector spinae plane block[Title/Abstract]) OR (erector spinae block[Title/Abstract])) OR (erector spinae muscle block[Title/Abstract])) OR (ESP[Title/Abstract])** |
| 5 | **((((((((((("Anesthesia, Epidural"[Mesh]) OR (Anesthesia, Peridural[Title/Abstract])) OR (Anesthesias, Peridural[Title/Abstract])) OR (Peridural Anesthesia[Title/Abstract])) OR (Peridural Anesthesias[Title/Abstract])) OR (Anesthesia, Extradural[Title/Abstract])) OR (Anesthesias, Extradural[Title/Abstract])) OR (Extradural Anesthesia[Title/Abstract])) OR (Extradural Anesthesias[Title/Abstract])) OR (Epidural Anesthesia[Title/Abstract])) OR (Anesthesias, Epidural[Title/Abstract])) OR (Epidural Anesthesias[Title/Abstract])** |
| 6 | ((((((((((((liver resection[Title/Abstract]) OR (liver surgery[Title/Abstract])) OR (hepatic resection[Title/Abstract])) OR (liver procedure[Title/Abstract])) OR (Hepatectomies[Title/Abstract])) OR (hepatectomy[Title/Abstract])) OR (laparoscopic liver surgery[Title/Abstract])) OR (laparoscopic liver resection[Title/Abstract])) OR (robotic liver resection[Title/Abstract])) OR (hepatic surgery[Title/Abstract])) OR (hepatic sectionectomy[Title/Abstract])) OR (liver transection[Title/Abstract])) OR (hepatic transection[Title/Abstract]) |
| 7 | #1 OR #2 OR #3 OR #4 OR #5 # |
| 8 | #7AND #6 |

Supplementary Table 2: Node-Splitting results

| Side | Direct Coef. | Std. Err. | Indirect Coef. | Std. Err. | Difference Coef. | Std. Err. | P>|z| |
| --- | --- | --- | --- | --- | --- | --- | --- |
| Resting pain score at 24h postoperatively | | | | | | | |
| A vs B | .8139687 | .5336296 | -.5989705 | .6760239 | 1.412939 | .8612601 | 0.101 |
| A vs C | .3842213 | .3807623 | 1.797142 | .7725274 | -1.412921 | .861266 | 0.101 |
| A vs G | - | - | - | - | - | - | - |
| B vs C | .983192 | .5586041 | -.429733 | .6555453 | 1.412925 | .8612655 | 0.101 |
| B vs D | 1.124654 | .7591723 | -.2859885 | 282.2374 | 1.410643 | 282.2385 | 0.996 |
| B vs E | .8308203 | .7507353 | -.5372073 | 630.5725 | 1.368028 | 630.5729 | 0.998 |
| D vs F | -.0714989 | .7324779 | -2.772497 | 631.2668 | 2.62391 | 631.2672 | 0.997 |
| Resting pain score at 48h postoperatively | | | | | | | |
| A vs B | 1.866294 | .2410262 | .1683101 | .2929384 | 1.697984 | .3793501 | 0.000 |
| A vs C | .1683105 | .1524862 | 1.866289 | .3473536 | -1.697979 | .3793502 | 0.000 |
| A vs G | - | - | - | - | - | - | - |
| B vs C | 9.95e-09 | .2501222 | -1.69798 | .2852114 | 1.69798 | .3793503 | 0.000 |
| B vs D | -.5647948 | .7683872 | -1.326741 | 283.4419 | .7619457 | 283.443 | 0.998 |
| B vs E | 1.591602 | .7773453 | -2.401464 | 630.3394 | 3.993066 | 630.3399 | 0.995 |
| D vs F | -.2421285 | .7486895 | -1.242104 | 632.1123 | .9999759 | 632.1127 | 0.999 |
| Resting pain score at 72h postoperatively | | | | | | | |
| A vs B | .0585026 | .2077441 | .5752783 | 158.1004 | -.5167756 | 158.1005 | 0.997 |
| B vs C | -.8907716 | .3339222 | -.3108347 | 172.7888 | -.579937 | 172.7891 | 0.997 |
| C vs D | -.1037493 | .2734222 | 1.664502 | .2734222 | -1.768252 | 0 | - |
| C vs E | 1.705649 | .3058541 | 1.664467 | .3058541 | .0411827 | 0 | - |
| C vs F | -.0131021 | .2191076 | 1.664651 | .2191076 | -1.677754 | 0 | - |
| Morphine consumption at 24h postoperatively | | | | | | | |
| A vs B | - | - | - | - | - | - | - |
| A vs C | 1.56892 | .2297541 | -.512366 | 183.861 | 2.081286 | 183.8611 | 0.991 |
| C vs D | .7160022 | .3200146 | -1.935344 | .3200146 | 2.651346 | 0 | - |
| C vs E | .7945634 | .2713987 | -3.137771 | .2713987 | 3.932335 | 0 | - |
| D vs F | .1300958 | .2214527 | -4.571298 | .2214527 | 4.701394 | 0 | - |
| Morphine consumption at 48h postoperatively | | | | | | | |
| A vs B | - | - | - | - | - | - | - |
| A vs C | 1.984893 | .245899 | -.4443869 | 182.8767 | 2.42928 | 182.8769 | 0.989 |
| C vs D | .7644454 | .282854 | -2.427167 | 287.7858 | 3.191612 | 287.7859 | .0.991 |
| C vs E | .2597189 | .2600278 | -3.969704 | .2600278 | 4.229423 | 0 | - |
| Dvs F * | .1119439 | .2188786 | -5.499906 | .2188786 | 5.61185 | 0 | - |

A:Epidural analgesia(EA); B:Continuous local anaesthetic infiltration(CLAI); C:Intrathecal Morphine(ITM); D:Erector spinae plane block(ESPB); E:Continuous ESPB (CESPB) F:Quadratus lumborum block(QLB); G:Continuous thoracic paravertebral block (CTPVB).

Supplementary Table 3: Values of surface under the cumulative ranking curve (SUCRA) and intervention ranking of all outcomes.

| Treatment | 24 h resting | | 48 h resting | | 24 h movement | | 48 h movement | | 24h morphine consumption | 24h morphine consumption | | |
| --- | --- | --- | --- | --- | --- | --- | --- | --- | --- | --- | --- | --- |
|  | SUCRA | Rank | SUCRA | Rank | SUCRA | Rank | SUCRA | Rank | SUCRA | Rank | SUCRA | Rank |
| EA | 85.1 | 1.9 | 84.8 | 1.9 | 93.7 | 1.1 | 99.7 | 1.0 | - | - | - | - |
| CLAI | 50.3 | 4.0 | 33.3 | 5.0 | - | - | - | - | - | - | - | - |
| ITM | 73.3 | 2.6 | 62.5 | 3.2 | 6.3 | 1.9 | 0.3 | 2.0 | - | - | - | - |
| ESPB | 25.8 | 5.5 | 56.7 | 3.6 | | - | - | - | - | - | - | - | - |
| CESPB | 35.5 | 4.9 | 2.8 | 6.8 | - | - | - | - | - | - | - | - |
| QLB | 33.3 | 5.0 | 65.4 | 3.1 | - | - | - | - | - | - | - | - |
| TPVB | 46.7 | 4.2 | 44.5 | 4.3 | - | - | - | - | - | - | - | - |

A:Epidural analgesia(EA);B:Continuous local anaesthetic infiltration(CLAI);C:Intrathecal Morphine(ITM);D:Erector spinae plane block(ESPB);E:Continuous ESPB (CESPB)F:Quadratus lumborum block(QLB);G:Continuous thoracic paravertebral block (CTPVB).
