# Supplementary material for: A comparison of regional anesthesia techniques for pain management in patients undergoing liver surgery: a network meta-analysis
Source: Front Med (Lausanne). 2025 Nov 28;12:1691322. doi: 10.3389/fmed.2025.1691322 (PMC12698550; doi:10.3389/fmed.2025.1691322)
Supplement: Supplementary file 2 [file Supplementary_file_1.docx]

Supplementary Figure1: Evidence network of eligible comparisons for a network meta-analysis of (A) Resting pain scores at 24 h postoperatively; (B) Resting pain scores 48 h postoperatively;(C) Resting pain scores 72 h postoperatively, (D) Movement pain scores at 24 h postoperatively;(E) Movement pain scores at 48 h postoperatively; (F) Morphine equivalent consumption 24 h postoperatively;(G) Morphine equivalent consumption 48 h postoperatively;(H) Morphine equivalent consumption 72 h postoperatively.

A B


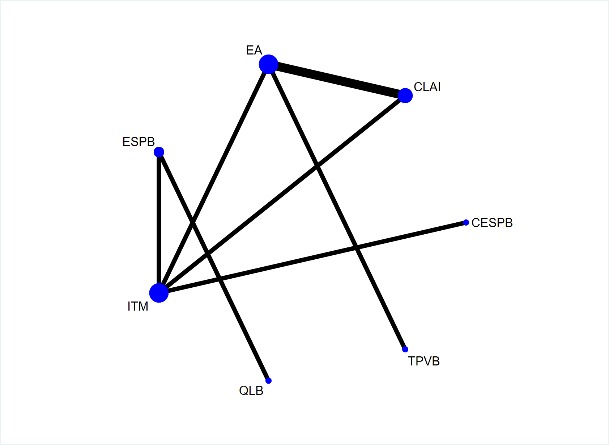

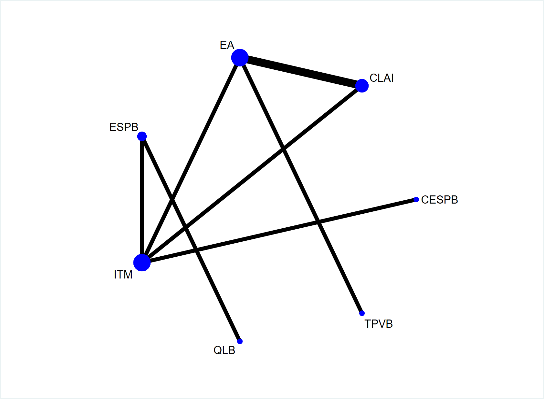


C D


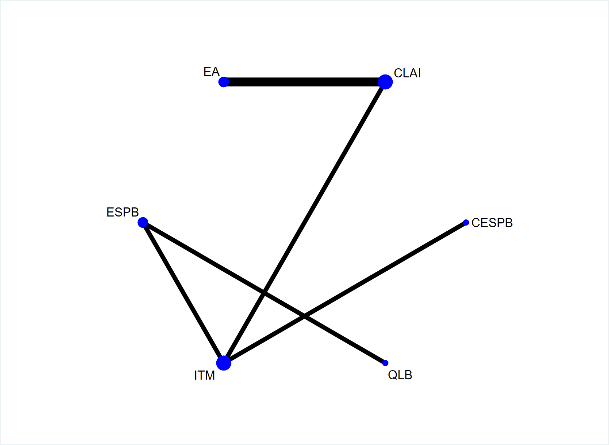

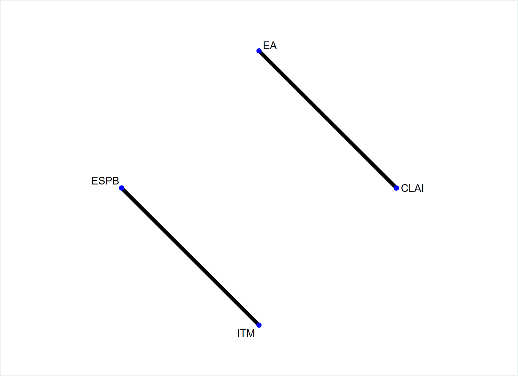


E F


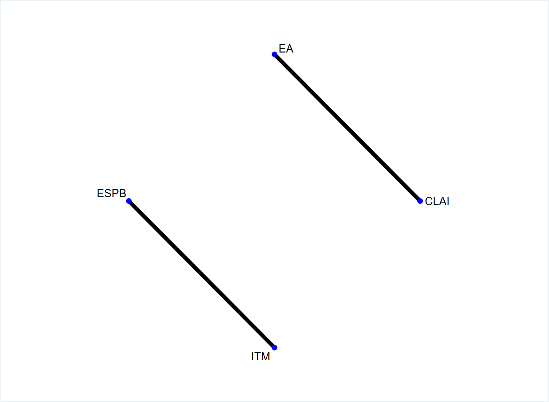

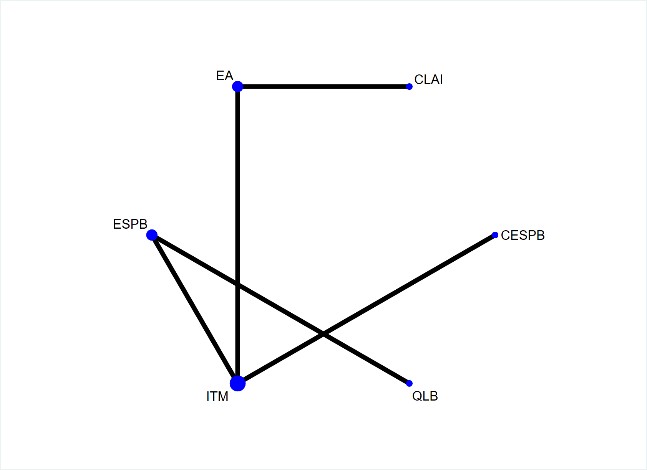


G H


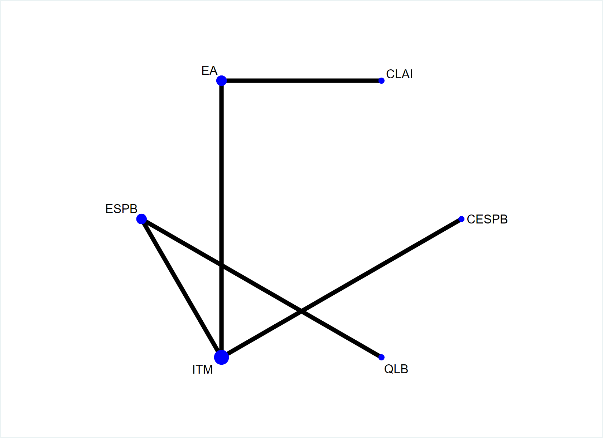

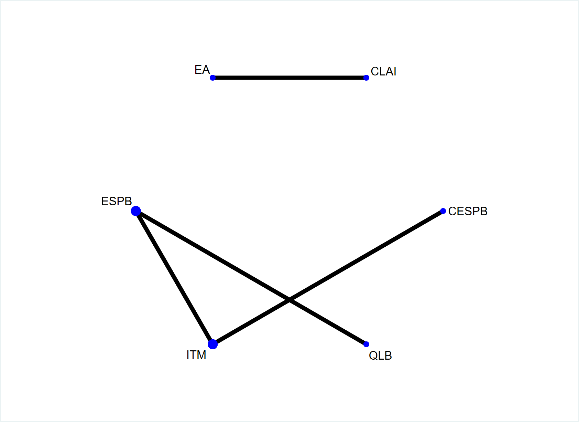


Supplementary Figure 2: The funnel plot of eligible comparisons for a network meta-analysis of (A) Resting pain scores at 24 h postoperatively; (B) Resting pain scores 48 h postoperatively;(C) Resting pain scores 72 h postoperatively, (D) Morphine equivalent consumption at 24 h postoperatively; (E) Morphine equivalent consumption at 48 h postoperatively; (F) Morphine equivalent consumption at 72 h postoperatively.

A


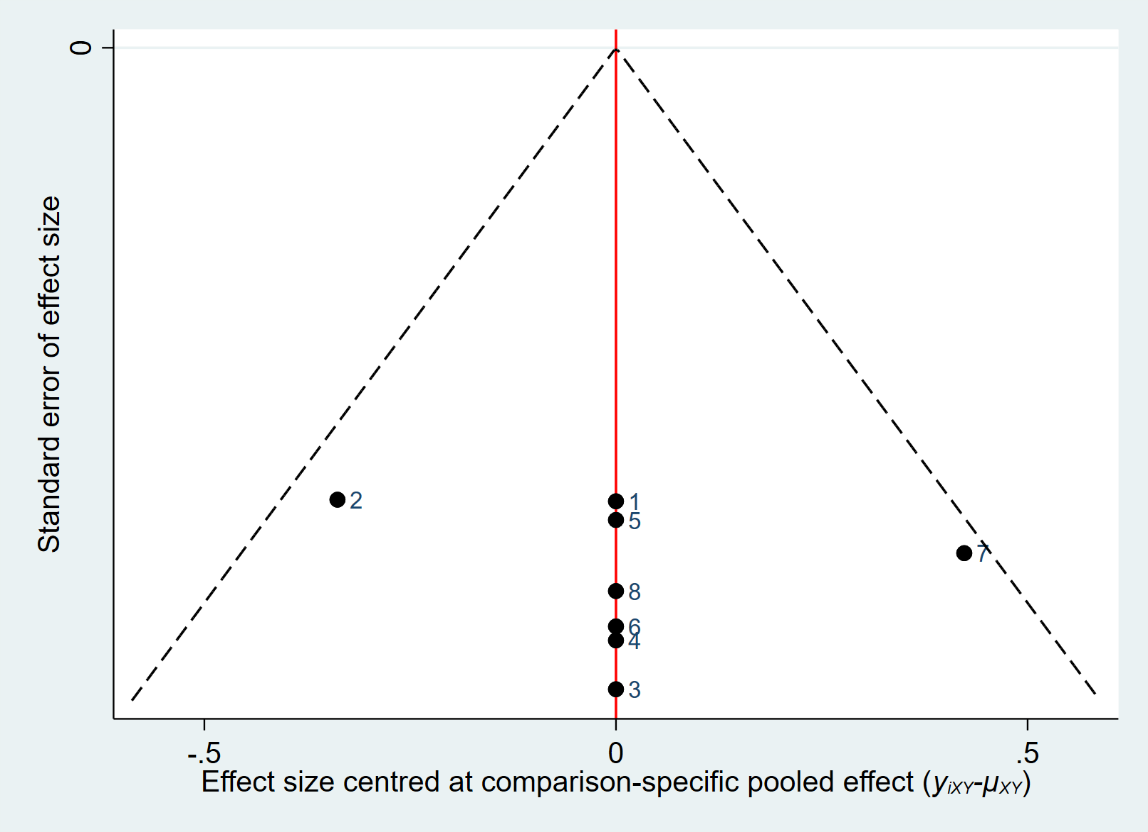


B

C


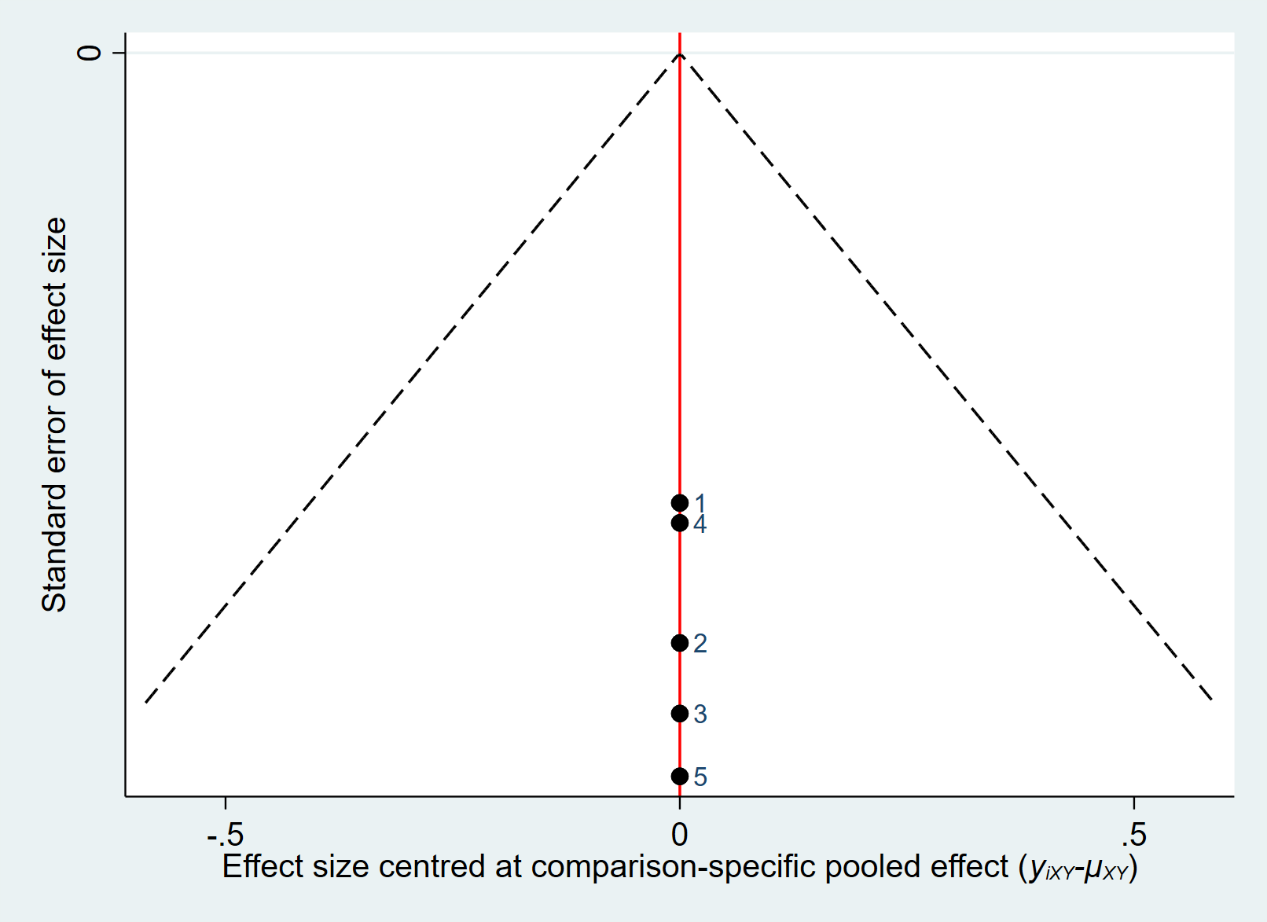


C

D

E

F

Supplementary Fig.3: Forest plot of eligible comparisons for a network meta-analysis of (A) Resting pain scores at 24 h postoperatively; (B) Resting pain scores 48 h postoperatively;(C) Resting pain scores 72 h postoperatively, (D) Morphine equivalent consumption 24 h postoperatively;(E) Morphine equivalent consumption 48 h postoperatively;(F) Morphine equivalent consumption 72 h postoperatively.

A

B

C

D

E

F
